# Social and health service-related factors associated with undiagnosed diabetes mellitus– a population-based survey in a highly urbanized Chinese setting

**DOI:** 10.1186/s12889-025-22048-0

**Published:** 2025-03-06

**Authors:** Paul K.M. Poon, King Wa Tam, Benjamin H.K. Yip, Roger Y. Chung, Eric K.P. Lee, Samuel Y.S. Wong

**Affiliations:** 1 Jockey Club School of Public Health and Primary Care, The Chinese University of Hong Kong, Sha Tin, New Territories, HKSAR, China; 2 CUHK Institute of Health Equity, HKSAR, China

**Keywords:** Diabetes mellitus, Preventive medicine, Public health

## Abstract

**Background:**

Undiagnosed diabetes mellitus (UDM) is associated with poorer health outcomes compared to diagnosed DM. We investigated factors associated with UDM in a highly urbanized Chinese setting to facilitate UDM detection.

**Methods:**

We analysed data from the cross-sectional Hong Kong Population Health Survey. We defined UDM by blood glucose and HbA1c levels and a negative history of self-reported doctor-diagnosed DM. We categorized diabetes status into UDM, incident DM (IDM, i.e. recently diagnosed) and individuals without diabetes and used multinomial logistic regression models to investigate the relationship between diabetes status and social and health service-related factors.

**Results:**

We included 98 IDM cases, 101 UDM cases, and 2,153 individuals without diabetes. Individuals aged 35–44 years (aOR 12.65, 95% C.I. 2.54–62.97) and those living in subsidized-sale housing (aOR 2.01, 95% C.I. 1.14–3.56) had a higher risk of UDM relative to not having diabetes, but not IDM. Males who were economically active (aOR 4.22, 95% C.I. 1.25–14.30), and males who did not have regular check-ups (aOR 3.05, 95% C.I. 1.16-8.00) had higher risks of UDM relative to not having diabetes, whereas males with a higher household income had a lower risk of UDM (aOR 0.94, 95% C.I. 0.89–0.99). Compared to individuals without diabetes, UDM cases had comparable physical activity levels but most were work- and transport-related rather than recreational.

**Conclusions:**

Compared to individuals without diabetes or IDM cases, economically active males, males without regular check-ups and males with lower household income had a higher risk of UDM. Targeted active DM screening can reduce UDM. However, further research on the benefits of different types of physical activity is needed.

## Background

Globally, diabetes mellitus (DM) affected approximately 425 million people in 2017 and is expected to reach 630 million by 2045 [1]. DM is a major cause of morbidity and mortality and leads to cardiovascular, renal, and neurological complications [2]. DM is often asymptomatic, especially in its early stages, when it can easily go unnoticed and undiagnosed [3]. Compared to diagnosed DM, undiagnosed DM (UDM) is associated with a higher risk of complication including diabetic nephropathy [4].

The prevalence of UDM varies across regions. A meta-analysis reported a range of 3.98–7.25% in 16 Eastern Mediterranean countries with different development indices, such as Iran, Pakistan, and Saudi Arabia [5]. Studies in the United States and Vietnam also showed similar single-digit prevalence rates, but the prevalence in Mexico was considerably higher at 18% [6–8]. An even higher UDM prevalence of 53.4% was seen in Bangladesh [9]. Such alarmingly high UDM prevalence is not only seen in low to middle-income regions but has also been observed in highly urbanized cities like Hong Kong (54.1%) [10].

Traditionally, DM screening recommendations adopt a risk-based approach, and various risk assessment tools have been developed, such as the Finnish Diabetes Risk Score and the Cambridge Risk Score [11–13]. These tools are useful for estimating the risk of DM in individuals by assessing biological factors (body mass index, waist circumference, age, sex), lifestyle factors (physical inactivity, unhealthy diet), medical history (history of hypertension, dyslipidaemia, or gestational diabetes), family history, ethnicity, and education level [14–16]. However, it is also important to identify the target groups to whom these risk assessment tools should be proactively applied to reduce the prevalence of UDM in the community. In addition to factors already considered in risk assessment tools, it is worthwhile to examine additional social and health service-related factors associated with UDM to allow targeted screening interventions. A German study found that individuals who lived alone and had not seen a doctor in the past year were more likely to have UDM, but these factors were not associated with diagnosed DM, highlighting the need for targeted screening in these populations [17]. Another study showed that having health insurance (Medicaid, private, or other) decreased the odds of undiagnosed prediabetes and having private health insurance specifically decreased the odds of UDM in the United States [6]. A systematic review of studies of African populations showed that urban residents had nearly double the risk of UDM compared to rural residents [18]. Social and health service-related factors associated with UDM differ across various social contexts and healthcare systems but studies in other highly urbanized areas are scarce. We analysed data from a population-based survey to investigate social and health service-related factors associated with UDM in a highly urbanized Chinese setting, and we hypothesized that working males in a metropolitan area would be at increased risk of UDM.

## Methods

### Study design and setting

The Population Health Survey (PHS) is conducted periodically by the Department of Health of the Hong Kong Special Administrative Region Government to examine the health status and behaviours of the general public. This survey was conducted between December 2014 and October 2015. The PHS is a population-based cross-sectional household survey. A sample of the Hong Kong general population was drawn using a systematic sampling method, by selecting a sample of representative domestic households at regular intervals from all addresses of permanent quarters in built-up areas and area segments in non-built-up areas in Hong Kong [10]. The sampling frame was based on census data and could produce a representative sample of the local population [10]. The sample included land-based non-institutional individuals aged 15 or above, excluding inmates of institutions, persons living on board vessels, foreign domestic helpers and visitors. The survey consisted of two parts, face-to-face interviews and blood tests. Further details on the survey methodologies can be found in the survey report [10].

Trained interviewers conducted face-to-face interviews to collect data on self-reported doctor-diagnosed chronic diseases, self-rated quality of life using the 12-item Chinese (Hong Kong) Short Form Health Survey (version 2) (SF-12v2 (HK)) [19], health-related lifestyle practices including the level of physical activity (PA) using the Global Physical Activity Questionnaire (GPAQ) developed by the WHO [20], health services utilization, and sociodemographic information. All interviewers received training sessions and a survey manual prior to fieldwork. Weekly debriefing sessions and further regular training sessions were arranged together with periodic on-site supervision and audit of completed questionnaires for quality control. The training and quality control were overseen by public health specialist doctors.

Blood tests were performed by trained staff, who were clinical laboratory personnel and supervised by medical doctors in designated centres, following the World Health Organization (WHO) STEPS Surveillance Manual [21]. The laboratory tests and results were reviewed by registered medical laboratory technologists and medical staff, including doctors and nurses, further reviewed all laboratory results. The same laboratory performed all the tests which was accredited by the Hong Kong Laboratory Accreditation. Moreover, there was daily internal quality control checking by the accredited laboratory and random on-site inspection of physical measurements and blood specimen collection by public health doctors from the Department of Health. All fasting blood samples were tested for both glucose and glycated haemoglobin (HbA1c) levels.

### Primary outcome

The primary outcome was DM status which was categorized as “UDM”, “IDM”, or “no diabetes”. We defined UDM cases as individuals who self-reported having no doctor-diagnosed DM in the face-to-face interview but were found to have an HbA1c level ≥ 6.5% or fasting blood glucose level ≥ 7.0 mmol/L in the blood tests. These cut-off values were based on the American Diabetes Association diagnostic guidelines [16]. For diagnosed DM cases, we used incident rather than prevalent cases. This approach helped keep the time interval between the DM diagnosis and data collection short and minimized temporal discrepancies in the data. This reduced the concern that behavioural and demographic data collected at the time of the survey might not reflect the situation at the time of their DM diagnosis. We defined incident DM (IDM) cases as individuals who answered “yes” to both questions: (1) “Have you ever been told by a doctor that you have diabetes?”; and (2) “Was the first time you were diagnosed or told by a doctor that you have the disease in the past 12 months?”. We excluded individuals who had been told by a doctor that they had diabetes for the first time more than 12 months before the survey (i.e. answering “yes” to question 1 and “no” to question 2) to improve the quality of the demographic and behavioural data reflecting the situation at the time of DM diagnosis and excluded those who answered “no” to question 1 and “yes” to question 2. Individuals without diabetes were those without self-reported doctor-diagnosed DM and with normal blood test results.

### Covariates

Covariates included demographic factors such as age group, sex, education level, marital status (married vs. single/widowed/divorced), and economic activity (active: having an income from a job/business vs. inactive: no active income from a job/business). Proxies for socioeconomic status (SES) including household income and housing type (public rental housing vs. subsidized-sale housing vs. private housing) were included. Public rental housing is government-subsidized housing for the lowest income group which only needs to pay a very low rental. Subsidized-sale housing is a home-ownership scheme for middle-income individuals to purchase housing units at a discounted price. Private housing is typically the choice for higher-income individuals. Additionally, lifestyle factors namely smoking status (ever smoker vs. non-smoker), and PA levels were also included. PA levels were assessed by the WHO’s GPAQ which collects information on activities at work, travelling, and recreational activities [20]. The total PA levels were calculated based on all types of activities. We analysed both the total PA levels and the levels of different types of PA. We adopted the WHO’s definition of an adequate PA level i.e. ≥600 metabolic equivalent of task (MET) minutes, or at least 150 min of moderate-intensity PA, or at least 75 min of vigorous-intensity PA per week [22]. Health service utilization was assessed by whether the respondents had undergone regular medical check-ups which were defined as any fixed or regular periodic medical check-ups, but there was no defined interval between two check-ups (i.e. regular check-ups vs. no regular check-ups). Other variables related to health status included self-rated quality of life based on the SF-12v2 (HK) and self-reported doctor-diagnosed hypertension.

### Statistical analyses

To test for any group differences across individuals without diabetes, IDM, and UDM cases, the chi-square test was performed on categorical and dichotomous variables and one-way ANOVA (analysis of variance) was performed on numerical variables. Multinomial logistic regression models were used to investigate the relationships between diabetes status and social and health service-related factors while adjusting for other covariates. We used multinomial logistic regression, which is an extension of logistic regression that generalizes to allow for the dependent variable to have more than two categories, rather than applying two separate binary logistic regression models. Two multinomial regression models were used to present the results from different perspectives. In the first model, the reference category was individuals without diabetes, which served to investigate risk factors associated with UDM and/or IDM compared to individuals without diabetes. In the second model, the reference category was IDM allowing for a direct comparison between UDM and IDM cases using a case-case study approach [17]. To further test our hypothesis, we particularly investigated the specific effect of social and health service-related factors on males by using interaction terms between sex and other variables (marital status, economic activity, household income, and regular medical check-ups) in the models to reflect differential effects between the sexes. Three individuals without diabetes were excluded from the analysis due to missing data on their income, and there were no missing data for the other variables. R software version 4.2.0 was used to perform the statistical analysis and the multinom function from the nnet package was used for fitting the multinomial logistic regression [23].

## Results

The PHS included 12,022 respondents from 5,435 domestic households. The response rate was 75.4% for the household survey. A total of 2,352 household survey respondents aged between 15 and 84 were further randomly selected to undergo blood tests. Among them, there were 98 IDM cases, 101 UDM cases, and 2,153 individuals without diabetes. The demographic characteristics of the participants are shown in Table 1. The overall mean (SD) age was 43.37 (17.10) years, which increased from individuals without diabetes to UDM and again to IDM cases (*p* < 0.001). UDM cases had the highest male-to-female ratio compared to the other two groups (*p* < 0.001). Similarly, a greater proportion of UDM cases lived in subsidized-sale housing (*p* = 0.004) (Table 1). UDM cases (3356 MET minutes) had an overall PA level in terms of MET minutes per week comparable to that of individuals without diabetes (3273 MET minutes). However, compared with individuals without diabetes, more of the reported PA in UDM cases were from work-related (Difference: 103 MET minutes per week, *p* = 0.951) and transport-related (Difference: 101 MET minutes per week, *p* = 0.151) activities, and less from recreational activities (Difference: -121 MET minutes per week, *p* = 0.136).

**Table 1:** Characteristics of individuals without diabetes, incident DM and undiagnosed DM cases

		No diabetes	Incident	Undiagnosed	*p*-value
	N	2153	98	101	
Age	Mean (SD)	41.94 (16.77)	59.01 (12.88)	58.76 (11.75)	< 0.001
	15–34	853 (39.6%)	6 (6.1%)	2 (2.0%)	< 0.001
	35–44	367 (17.0%)	6 (6.1%)	11 (10.9%)	
	45–54	372 (17.3%)	19 (19.4%)	23 (22.8%)	
	55–64	325 (15.1%)	36 (36.7%)	23 (22.8%)	
	65–74	182 (8.5%)	18 (18.4%)	35 (34.7%)	
	75+	54 (2.5%)	13 (13.3%)	7 (6.9%)	
Sex	Male	1011 (47.0%)	37 (37.8%)	64 (63.4%)	< 0.001
	Female	1142 (53.0%)	61 (62.2%)	37 (36.6%)	
Self-rated Quality of Life	Mean (SD)	3.00 (0.89)	2.51 (0.89)	2.68 (0.84)	< 0.001
Hypertension	No	1923 (89.3%)	40 (40.8%)	74 (73.3%)	< 0.001
	Yes	230 (10.7%)	58 (59.2%)	27 (26.7%)	
Ever smoke	No	1918 (89.1%)	80 (81.6%)	82 (81.2%)	0.005
	Yes	235 (10.9%)	18 (18.4%)	19 (18.8%)	
Regular medical check-ups	No	1355 (62.9%)	49 (50.0%)	61 (60.4%)	0.033
	Yes	798 (37.1%)	49 (50.0%)	40 (39.6%)	
Housing type	Public rental	684 (31.8%)	44 (44.9%)	30 (29.7%)	0.004
	Subsidised-sale	387 (18.0%)	17 (17.3%)	29 (28.7%)	
	Private	1082 (50.3%)	37 (37.8%)	42 (41.6%)	
Education	Primary or below	276 (12.8%)	33 (33.7%)	35 (34.7%)	< 0.001
	Secondary	1051 (48.8%)	55 (56.1%)	45 (44.6%)	
	Post-secondary	826 (38.4%)	10 (10.2%)	21 (20.8%)	
Currently married	No	938 (43.6%)	31 (31.6%)	14 (13.9%)	< 0.001
	Yes	1215 (56.4%)	67 (68.4%)	87 (86.1%)	
Economically active	No	772 (35.9%)	55 (56.1%)	51 (50.5%)	< 0.001
	Yes	1381 (64.1%)	43 (43.9%)	50 (49.5%)	
Household income	Mean (SD)	13559.10 (13350.51)	8877.55 (11473.93)	8908.42 (9857.66)	< 0.001
	$0	423 (19.6%)	21 (21.4%)	17 (16.8%)	< 0.001
	$1 - $5,999	380 (17.6%)	37 (37.8%)	32 (31.7%)	
	$6,000 - $12,499	407 (18.9%)	18 (18.4%)	28 (27.7%)	
	$12,500 - $19,999	420 (19.5%)	9 (9.2%)	13 (12.9%)	
	$20,000 - $29,999	274 (12.7%)	6 (6.1%)	7 (6.9%)	
	$30,000 or above	249 (11.6%)	7 (7.1%)	4 (4.0%)	
Meeting WHO recommended PA level	No	246 (11.4%)	22 (22.4%)	8 (7.9%)	0.002
	Yes	1907 (88.6%)	76 (77.6%)	93 (92.1%)	
Total MET per week (minutes)	Mean (SD)	3273.08 (4081.99)	2698.16 (4010.53)	3356.04 (4330.36)	0.383
Work-related MET per week (minutes)	Mean (SD)	911.28 (3417.69)	875.51 (3647.95)	1014.06 (3159.51)	0.951
Transport-related MET per week (minutes)	Mean (SD)	1813.18 (1931.68)	1443.06 (1456.68)	1914.65 (2349.18)	0.151
Recreational MET per week (minutes)	Mean (SD)	548.62 (1017.77)	379.59 (676.77)	427.33 (656.52)	0.136
Sedentary time per day (minutes)	Mean (SD)	412.52 (161.41)	369.08 (165.62)	402.48 (155.73)	0.030

SD; standard deviation. PA: physical activity. MET: metabolic equivalent of task. N; the number of observations. The *p*-values indicate the level of significance of chi-square tests for categorical/dichotomous variables, and that of one-way ANOVA for numerical variables

The first multinomial logistic regression model (Table 2) showed that, relative to individuals without diabetes, factors independently associated with UDM, but not IDM, include being in the 35–44 age group (aOR 12.65, 95% C.I. 2.54–62.97) and living in subsidized-sale housing (aOR 2.01, 95% C.I. 1.14–3.56). The interactions between being male and factors including being economically active (aOR 4.22, 95% C.I. 1.25–14.30) and not undergoing regular check-ups (aOR 3.05, 95% C.I. 1.16-8.00) were associated with a higher risk of UDM relative to not having diabetes, but not IDM. The interaction between being male and higher household income (aOR 0.94, 95% C.I. 0.89–0.99) was associated with a lower risk of UDM relative to not having diabetes, but not IDM (Table 2).

**Table 2:** Multinomial logistic regression: incident DM cases and undiagnosed DM cases vs. individuals without diabetes

Reference level: No diabetes (*N* = 2153)		Incident DM *N* = 98		Undiagnosed DM *N* = 101	
		Odds ratio	95% CI	Odds ratio	95% CI
Age	15–34	1.000 (ref)		1.000 (ref)	
	35–44	1.677	(0.493, 5.698)	12.653**	(2.543, 62.970)
	45–54	4.126**	(1.453, 11.720)	28.888***	(5.916, 141.052)
	55–64	7.644***	(2.730, 21.405)	20.823***	(4.166, 104.073)
	65–74	4.998**	(1.609, 15.526)	59.744***	(11.827, 301.795)
	75+	9.957***	(2.897, 34.214)	38.712***	(6.504, 230.402)
Male		0.611	(0.203, 1.845)	3.356	(0.897, 12.562)
Self-rated Quality of Life		0.835	(0.638, 1.093)	0.833	(0.646, 1.074)
Meeting WHO recommended PA		0.423**	(0.239, 0.750)	1.631	(0.749, 3.554)
Hypertension		6.077***	(3.673, 10.054)	1.187	(0.703, 2.003)
Ever smoke		2.871**	(1.475, 5.589)	1.284	(0.704, 2.342)
Housing type	Public rental	1.000 (ref)		1.000 (ref)	
	Subsidized-sale	0.646	(0.342, 1.218)	2.010*	(1.135, 3.560)
	Private	0.774	(0.460, 1.303)	1.173	(0.686, 2.007)
Education	Primary or below	1.000 (ref)		1.000 (ref)	
	Secondary	1.223	(0.702, 2.132)	0.656	(0.384, 1.120)
	Post-secondary	0.490	(0.193, 1.245)	1.171	(0.572, 2.395)
Currently married		0.859	(0.466, 1.583)	1.672	(0.730, 3.831)
Economically active		1.352	(0.620, 2.948)	0.749	(0.279, 2.009)
Household income (per $1000)		0.983	(0.936, 1.033)	0.991	(0.948, 1.036)
Regular medical check-ups		0.834	(0.468, 1.487)	1.801	(0.872, 3.723)
Sedentary time per day (hour)		0.956	(0.879, 1.040)	1.067	(0.983, 1.157)
Male × currently married		0.545	(0.186, 1.597)	0.981	(0.281, 3.422)
Male × economically active		0.584	(0.175, 1.944)	4.223*	(1.247, 14.302)
Male × household income		1.043	(0.984, 1.106)	0.937*	(0.885, 0.992)
Male × no regular check-ups		0.513	(0.204, 1.292)	3.049*	(1.160, 8.000)

PA: physical activity. CI: confidence intervals. All of the above variables and interaction terms were included as covariates in the multinomial regression model. Married denotes whether the individual is married or not (single/widowed/divorced). Economically active denotes whether the individual has income from a job or business. The McFadden R^2^ is 0.222 and 0.363 for the incident DM and undiagnosed DM models respectively

* *p* < 0.05; ** *p* < 0.01; *** *p* < 0.001

The second multinomial logistic regression model (Table 3) presented another perspective on the comparisons– a direct case-case comparison between UDM and IDM cases. Compared to IDM, UDM cases were more likely to be male (aOR 5.49, 95% C.I. 1.04–28.96), to attain an adequate overall level of PA (aOR 3.85, 95% C.I. 1.51–9.80), and to live in subsidized-sale housing (aOR, 3.11 95% C.I. 1.37–7.09), but less likely to have a history of hypertension (aOR, 0.20 95% C.I. 0.10–0.39). The interactions between being male and factors including being economically active (aOR 7.24, 95% C.I. 1.37–38.19) and not undergoing regular check-ups (aOR 5.95, 95% C.I. 1.64–21.74) were associated with a higher risk of UDM relative to IDM (i.e. an increased risk of being undiagnosed among DM cases). The interaction between being male and higher household income (aOR 0.90, 95% C.I. 0.83–0.97) was associated with a lower risk of UDM relative to IDM.

**Table 3:** Multinomial logistic regression: individuals without diabetes and undiagnosed DM cases vs. incident DM cases

Reference level: Incident DM (*N* = 98)		Undiagnosed DM *N* = 101		No diabetes *N* = 2153	
		Odds ratio	95% CI	Odds ratio	95% CI
Age	15–34	1.000 (ref)		1.000 (ref)	
	35–44	7.549*	(1.015, 56.126)	0.596	(0.175, 2.027)
	45–54	7.003*	(1.064, 46.096)	0.242**	(0.085, 0.688)
	55–64	2.725	(0.410, 18.104)	0.131***	(0.047, 0.366)
	65–74	11.957*	(1.693, 84.460)	0.200**	(0.064, 0.622)
	75+	3.889	(0.464, 32.580)	0.100***	(0.029, 0.345)
Male		5.491*	(1.041, 28.957)	1.636	(0.542, 4.935)
Self-rated Quality of Life		0.998	(0.697, 1.429)	1.198	(0.915, 1.569)
Meeting WHO recommended PA		3.853**	(1.514, 9.802)	2.362**	(1.334, 4.182)
Hypertension		0.195***	(0.097, 0.393)	0.165***	(0.099, 0.272)
Ever smoke		0.447	(0.188, 1.062)	0.348**	(0.179, 0.678)
Housing type	Public rental	1.000 (ref)		1.000 (ref)	
	Subsidized-sale	3.114**	(1.367, 7.093)	1.549	(0.821, 2.922)
	Private	1.516	(0.736, 3.122)	1.292	(0.767, 2.175)
Education	Primary or below	1.000 (ref)		1.000 (ref)	
	Secondary	0.536	(0.255, 1.124)	0.818	(0.469, 1.425)
	Post-secondary	2.388	(0.762, 7.479)	2.040	(0.804, 5.178)
Currently married		1.947	(0.719, 5.273)	1.164	(0.632, 2.146)
Economically active		0.554	(0.162, 1.890)	0.740	(0.339, 1.614)
Household income (per $1000)		1.008	(0.944, 1.076)	1.017	(0.968, 1.069)
Regular medical check-ups		2.161	(0.880, 5.306)	1.199	(0.673, 2.139)
Sedentary time per day (hour)		1.115	(0.996, 1.249)	1.046	(0.961, 1.137)
Male × currently married		1.800	(0.365, 8.880)	1.835	(0.626, 5.377)
Male × economically active		7.236*	(1.371, 38.189)	1.714	(0.514, 5.709)
Male × household income		0.898**	(0.829, 0.974)	0.958	(0.904, 1.016)
Male × no regular check-ups		5.952**	(1.637, 21.739)	1.949	(0.773, 4.902)

PA: physical activity. CI: confidence intervals. All of the above variables and interaction terms were included as covariates in the multinomial regression model. Married denotes whether the individual is married or not (single/widowed/divorced). Economically active denotes whether the individual has income from a job or business. The McFadden R^2^ is 0.222 and 0.363 for the undiagnosed DM and no diabetes models respectively. The McFadden R^2^ is 0.363 and 0.222 for the undiagnosed DM and no diabetes models respectively

* *p* < 0.05; ** *p* < 0.01; *** *p* < 0.001

## Discussion

Both diagnosed DM and UDM may share similar biological or genetic etiological factors but UDM is associated with a poorer cardiovascular risk profile [24]. Compared to individuals without diabetes, we found that people aged 35–44 years, those living in subsidized-sale housing, economically active males, males without regular medical check-ups, and males with a lower household income all had higher risks of UDM relative to not having diabetes, but not incident DM. In addition, we used a case-case study approach to demonstrate that, among patients with DM, these factors also increased the risk of the disease being undiagnosed. In our study, economically active males were a group with a higher risk of UDM. The risk of UDM starts to increase at 35–44 years of age, which is earlier than the time when an increased risk of IDM was observed (Table 2). This could indicate that many DM cases in this younger middle-aged group might be undiagnosed. These findings are largely consistent with other studies showing that males and younger individuals had an increased risk of UDM, compared to females and older individuals respectively [17, 25].

Previous studies have shown that receiving healthcare in the past year and having a routine place to receive healthcare were associated with a lower risk of UDM [26, 27]. Similarly, we demonstrated that undergoing regular medical check-ups among males could counter their risk of UDM. This was also reflected in our study, as people with doctor-diagnosed hypertension were at a lower risk of UDM because patients with hypertension in Hong Kong would be offered routine DM screening by their attending doctors [28]. This finding is consistent with the findings of another study showing a lower risk of UDM among patients with hypertension [26]. On the other hand, the working hours in Hong Kong are among the longest in the world [29], which could deprive the working population of the time and opportunity to undergo regular medical check-ups. It is important that public health screening programmes actively reach out to this subgroup, for instance, via a workplace-based DM screening programme. Economically active males in their thirties and forties can be the target group for proactive DM screening.

Moreover, we demonstrated that SES further delineated the group most vulnerable to UDM (Table 1). We showed that higher household income among males decreased the risk of UDM. In our study, nearly 60% of UDM cases had a monthly household income below the median wage of the Hong Kong population (HK$14,800 ~ USD1,897 in 2014 [30]) compared to 36.5% among individuals without diabetes. The overall mean income of UDM cases (HK$8,908 ~ USD 1,142) was marginally higher than that of IDM cases (HK$8,877 ~ USD 1,138) but much lower than that of individuals without diabetes (HK$13,559 ~ USD1,738). In other words, UDM cases were in the lower working class with regard to household income. A 2019 government report showed that elementary workers were more prone to long working hours, including cleaners, security guards, building caretakers, messengers, delivery workers, couriers, dishwashers, freight handlers, lift operators, labourers/general workers, hand packers, and card/pamphlet distributors [31]. It was also found that the prevalence of UDM among professional drivers was 8.1% which was nearly double the prevalence in the general population [10, 32]. Similarly, it has been observed in England that people in manual occupations were associated with a higher risk of UDM [33]. The distribution of housing types further revealed the SES of our UDM cases. Compared to individuals without diabetes and IDM cases, more UDM cases were living in subsidized-sale housing (Table 1), which is generally less expensive than private housing but still places a greater financial burden on owners than public rental housing. In Hong Kong, housing costs are notoriously high and constitute the largest expenses for most citizens. After all, individuals with UDM were typically economically active, did not have a high household income, and might have a higher financial burden than people living in public rental housing. These could result in longer working hours for these people. On the other hand, the IDM cases might have similar income levels as the UDM cases but they had less financial burden from housing costs which might allow them to work shorter hours.

Intriguingly, we found that, compared to individuals without diabetes, adequate PA had a protective effect on IDM, but not UDM (Table 2), and those with adequate PA were more likely to have no diabetes or UDM than IDM (*p* < 0.01) (Table 3). Findings from a recent study in the United States suggested that different types of PA might have different health effects on the risk of DM [34]. Our study also revealed that, although UDM cases had PA levels comparable to those of individuals without diabetes in terms of total MET minutes per week, the composition of PA types differed (Table 1). A total of 87.2% of the PA in individuals with UDM was either work- or transport-related, with less than 13% from recreational activities compared to 16.7% recreational PA in individuals without diabetes. Although the difference in proportion did not reach statistical significance in our study, further research is warranted to evaluate the effects and confounders (e.g., overall healthier lifestyle in people with more recreational exercise) on the associations between different types of PA and the risk of DM.

### Strengths and limitations

This study has several strengths. The PHS was population-based and the sampling was based on government census data ensuring a representative sample of the general population. All laboratory tests were performed in accredited laboratories. The study employed established diagnostic criteria for diabetes, ensuring that the findings are relevant and comparable to other studies. However, there are a few limitations to consider. The cross-sectional study design restricts the ability to infer a temporal cause-and-effect relationship between risk factors and UDM. Second, the diagnosis of DM was mainly self-reported and might not be as accurate as medical records. Nevertheless, the overall prevalence of DM reported in the PHS was comparable to the prevalence reported in official health records in another local study [35]. Third, UDM was defined by one blood test result for either fasting glucose or HbA1c, while two separate abnormal results are usually required for a definitive DM diagnosis in clinical settings [36]. Fourth, the PHS was not specifically designed for investigating UDM and data were not collected for variables such as family history of DM. Last, the fasting status of participants was self-reported. However, this approach was reasonably reliable because staff in the health examination centres were trained to verify the fasting status just before blood collection by asking the participants about the time of their last food intake.

## Conclusions

Individuals at a higher risk of undiagnosed diabetes were men who were economically active, had lower household income, and did not undergo regular medical check-ups. DM screening programmes should target this vulnerable group to reduce the prevalence of UDM in the community. However, further research is needed to delineate the health benefits of different types of physical activity for preventing DM.

## Data Availability

Data access and use were approved by the Department of Health of the Hong Kong Special Administrative Region Government. Data access may be obtained by sending a request via email to the Department of Health of the Hong Kong Special Administrative Region Government (enquiries@dh.gov.hk).

## Abbreviations

aOR: Adjusted odds ratio
DM: Diabetes mellitus
GPAQ: Global Physical Activity Questionnaire
HbA1c: Glycated haemoglobin
IDM: Incident diabetes mellitus
MET: Metabolic equivalent of task
PA: Physical activity
SES: Socioeconomic status
UDM: Undiagnosed diabetes mellitus
